# Sex- and age-stratified pharmacovigilance of methotrexate-associated adverse events in pediatric leukemia: a FAERS-based study

**DOI:** 10.3389/fmed.2026.1948388

**Published:** 2026-09-28

**Authors:** Min Lai, Shiyu Li, Wenqing Wang, Dan Liu, Wei Yang, Luping Wen, Junbin Huang, Hongman Xue

**Affiliations:** 1 Pediatric Hematology Laboratory, Division of Hematology/Oncology, Department of Pediatrics, The Seventh Affiliated Hospital of Sun Yat-sen University, Shenzhen, China; 2 Department of Pediatrics, Nanfeng County People’s Hospital, Fuzhou, Jiangxi, China; 3 Department of Pediatrics Surgery, The Seventh Affiliated Hospital of Sun Yat-sen University, Shenzhen, China; 4 Department of Pharmacy, The Seventh Affiliated Hospital of Sun Yat-sen University, Shenzhen, China

**Keywords:** FAERS, methotrexate, pediatric leukemia, pharmacovigilance, signal detection

## Abstract

**Background:**

Pediatric leukemia (PL) is a common malignant tumor in childhood. Methotrexate (MTX) is a widely used chemotherapeutic agent for the treatment of this disease. This study was based on the FAERS database and explored the adverse reaction signals of MTX in the treatment of PL.

**Methods:**

This study retrieved reports of adverse drug events (ADEs) related to MTX in the treatment of PL from the FAERS database (Q1 2018–Q1 2025). The disproportionality approach, including Reporting Odds Ratio (ROR), Proportional Reporting Ratio (PRR), Bayesian Confidence Propagation Neural Network (BCPNN), and Multi-item Gamma Poisson Shrinker (MGPS), was employed to detect ADE signals. Subgroup analyses were conducted by gender and age groups (infants: <1 year; toddlers: 1–3 years; preschoolers: 4–6 years; school-age: 7–10 years; adolescents: 11–17 years), and time-to-onset of ADEs was analyzed using Weibull distribution fitting.

**Results:**

A total of 2,907 reports of ADEs associated with MTX treatment for PL were included. Among these reports, males accounted for the highest proportion (58.3%); the main outcome of ADEs was hospitalization (41.9%). At the SOC level, nervous system disorders accounted for the largest number of reports; at the PT level, febrile neutropenia had the highest report count. Among gender-related ADE signals, confusional state showed greater reporting disproportionality in males. In the age-stratified analysis, the leading PTs varied across age groups; 6 signals were detected in all age groups, and reports describing events within 0–30 days accounted for the highest proportion (72.1%).

**Conclusion:**

This study identified prominent reporting signals for nervous system disorders, with febrile neutropenia, neurotoxicity, and mucositis among the most frequently reported PTs. Reports involving males and adolescents accounted for larger proportions, and 72.1% of reports with available onset data described events within 30 days. These findings are hypothesis-generating pharmacovigilance signals and do not establish incidence, comparative clinical risk, or causality.

## 1 Introduction

Pediatric leukemia (PL) is a cancer that originates in hematopoietic stem cells in the bone marrow. It is the most common type of childhood cancer (1). PL can be divided into two groups. The first is acute lymphoblastic leukemia (ALL), the most common type. The disease progresses rapidly but responds well to chemotherapy, leading to favorable outcomes (2–4). Second is acute myeloid leukemia (AML). Leukemia cells originate from myeloid hematopoietic stem cells. Some subtypes are more aggressive and require complex treatment plans (5, 6). Additionally, chronic myeloid leukemia (CML) and chronic lymphocytic leukemia (CLL) are extremely rare in children, together accounting for less than 1% of cases (7, 8). Methotrexate (MTX) is a key chemotherapeutic agent for treating childhood leukemia, playing a vital role in improving patient prognosis (9). However, given disease heterogeneity and challenges in dose adjustment and adverse reaction management during clinical application, its optimal utilization strategy in personalized treatment remains to be further explored.

MTX is a folate antagonist chemotherapy drug that has played a crucial role in treating various malignancies since the 1950s. It occupies an indispensable position in PL (10, 11). The drug's primary mechanism is to competitively inhibit dihydrofolate reductase, thereby preventing the conversion of dihydrofolate to the physiologically active form, tetrahydrofolate (12). Tetrahydrofolate is a vital coenzyme in the synthesis of nucleic acid precursors. Blocking its production severely inhibits the synthesis of DNA and RNA within tumor cells. This stops abnormally proliferating leukemia cells from dividing and growing as they should (13, 14). However, MTX lacks strict cell selectivity in its inhibition of dihydrofolate reductase. Leukemia cells have a high proliferation rate and require nucleic acid synthesis. However, normal cells with high proliferation rates, such as oral mucosal epithelial cells, bone marrow hematopoietic cells, and neurons, also depend on folate metabolism to maintain their function. MTX attacks both tumor cells and normal cells, and this lack of selectivity underlies its adverse reactions (15, 16). Including mucosal inflammation, gastrointestinal reactions, liver function impairment and bone marrow suppression,which result in prolonged hospital stays and increased disease burden (17, 18).

This study used data from the FAERS database to analyze the ADEs associated with MTX treatment for PL. It provides the first systematic characterization of the complete spectrum of adverse drug events associated with MTX therapy in PL.Through statistical analysis of data from the first quarter of 2018 to the first quarter of 2025, we used various data mining methods to characterize sex- and age-related differences in the reporting patterns and signal strength of MTX-associated ADEs in PL. We also analyzed the timing of adverse reactions. Ultimately, this study aims to provide clinicians with scientific data and practical references to help them develop personalized medication plans, identify subgroups warranting enhanced pharmacovigilance, and intervene promptly in cases of adverse reactions, thereby enhancing the safety and effectiveness of MTX treatment for PL.

## 2 Materials and methods

### 2.1 Data extraction

The original data of this study were derived from the United States (US) FAERS database. This database collects voluntarily reported adverse drug event (ADE) reports from healthcare providers, patients, and other relevant parties. It is updated quarterly, and data in ASCII-2 or XML format can be obtained for free. It was commonly used for detecting adverse event signals of marketed drugs. The data in the FAERS database were standardized using the Medical Dictionary for Regulatory Activities (MedDRA). Each report was coded using “Preferred Term (PT)” and “System Organ Class (SOC)”, and the standardized MedDRA was applied to identify PTs and SOCs related to MTX. Among them, SOCs were grouped according to etiology, location of occurrence, or purpose; PT was a specific term describing a single medical concept, and each PT corresponded to at least one SOC.

For case identification, we used the INDI_PT field in the FAERS indication information (INDI) to identify leukemia-related cases. We filtered records where INDI_PT contained “LEUKAEMIA” and restricted the age range to 0–17 years to obtain pediatric leukemia-related cases. Second, regarding drug identification, we matched methotrexate and its brand name, Trexall, from the drug information and restricted the drug role to Primary Suspect (PS). Specifically, we filtered relevant drug records based on ROLE_COD = “PS” and set drugEffect = “PS” during the easyFAERS::AFun() data extraction process. Consequently, this study included reports in which methotrexate was the primary suspect drug; reports where the drug role was “Secondary Suspect” (SS) or “Concomitant” (C) were not included in the analysis as MTX-related reports. The inclusion criteria of this study were: adverse event reports related to MTX used for the treatment of PL within the specified study period. The data exclusion criteria were: ① excluding the IDs of MTX-related adverse event reports that were officially deleted, duplicated, or missing by the FDA; ② excluding reports with inaccurate gender, reporter, serious outcome, and outcome; ③ excluding reports with unclear age or those aged 18 years and above; ④ for duplicate cases, they are identified by comparing the FDA_DT values under the same CASEID, and the report corresponding to the most recent FDA_DT is retained; when both the CASEID and FDA_DT are the same, the report with the higher PRIMARYID is retained to prevent duplicate reporting of the same case from affecting the analysis results. From the annual FAERS data, there were 22,249,476 patient demographic (DEMO) records before removing duplicates, and 18,627,667 after removing duplicates; there were 67,864,973 drug use record (DRUG) entries after removing duplicates; there were 54,514,632 drug adverse reaction information (REAC) records after removing duplicates; there were 55,020,348 treatment duration (THER) entries after removing duplicates; there were 19,312,635 report source (RPSR) records after removing duplicates; there were 18,627,667 patient outcome (OUTC) records after removing duplicates. Finally, 2,907 ADE reports of MTX in the treatment of PL were included.

### 2.2 Data mining

This study employed the disproportionality approach to detect signals of ADEs. At its core, this methodology relies on a disproportionality fourfold table to contrast the frequencies of specific drugs and events of interest against their corresponding background frequencies **(**Table 1**)**. Four distinct methods were utilized for this purpose: the reporting odds ratio (ROR), proportional reporting ratio (PRR), the Bayesian Confidence Propagation Neural Network method (BCPNN)and multi-item gamma Poisson shrinker (MGPS).

**Table 1:** Two-by-two contingency table.

Drug	Number of target event reports	Number of other incident reports	Total
Target drug	a	b	a + b
Other drugs	c	d	c + d
Total	a + c	b + d	a + b + c + d

The calculation formula for the ROR was: $\mathrm{ROR}=\frac{a/c}{b/d}$. The 95% confidence interval (CI) of the ROR value was calculated as: $95\text{\%}\mathrm{CI}=e^{\ln(\mathrm{ROR})\;\pm \;1.96\mathrm{SE}(\mathrm{lnROR})}$. When a ≥ 3 and the lower limit of the 95% CI of the ROR value was greater than 1, it indicated that the ADE reports of the relevant drug were more frequent, which suggested the existence of a potential safety signal. The calculation formulas for the PRR were as follows: $\mathrm{PRR}=\frac{a/(a+b)}{c/(c+d)}$, and $\chi^{2}=\frac{\;(\mathrm{ad}\;\text{–}\;\mathrm{bc}{)}^{2}(a+b+c+d)}{(a+b)(c+d)(a+c)(b+d)}$. When a ≥ 3, PRR ≥ 2, and *χ*^2^ ≥ 4, it indicated that the reporting proportion of ADEs associated with the drug was relatively high, and there might be a potential signal. The calculation formula for the BCPNN was: $\mathrm{IC}=\mathrm{lo}g_{2}\frac{a(a+b+c+d)}{(a+b)(a+c)}$. When IC_025_ > 0, it indicates that the rate of ADE reports for the drug is higher than expected. The calculation formula for the empirical bayesian geometric mean (EBGM) was: $\mathrm{EBGM}=\frac{a(a+b+c+d)}{(a+c)(a+b)},\;\mathrm{EBG}M_{05}=e^{\ln(\mathrm{EBGM})}\text{-}2\sqrt{1.64\frac{1}{a}+\frac{1}{b}+\frac{1}{c}+\frac{1}{d}}$ As the EBGM is a key statistic derived from the MGPS algorithm—specifically representing the geometric mean of the posterior distribution generated by MGPS—when EBGM_05_ > 2 and a > 0, it indicated a stronger association between the drug and ADEs (Supplementary Table S1).

These four algorithms were comprehensively applied to minimize the potential bias inherent in a single analytical method, thereby enabling more comprehensive and reliable identification of ADE signals. When the number of ADE reports was ≥3, the lower limit of the 95% CI for ROR was >1, the PRR was ≥2, the *χ*^2^ value was ≥4, the IC_025_ was >0, the EBGM_05_ was >2 and a > 0, an ADE signal was determined. Higher signal-strength values indicate greater reporting disproportionality rather than incidence or causality. The analyses were exploratory signal-detection procedures; sex-, age-, and time-stratified comparisons were descriptive, and no exposure-adjusted incidence, risk estimates, or causal-effect calculations were performed.

### 2.3 Baseline characteristics of the study subjects

To characterize the basic features of ADEs associated with MTX in the treatment of PL, an analysis was conducted on ADE reports of MTX for PL treatment based on the FAERS data from the first quarter of 2018 to the first quarter of 2025. The included ADE reports were statistically analyzed by dimensions of gender, body weight, occupation, outcome, and reporting country.

### 2.4 Analysis of detection at the SOC levels and PT levels

To investigate the SOC and PT levels involved in ADEs of MTX in the treatment of PL, relevant data from the first quarter of 2018 to the first quarter of 2025 in the FAERS database were used, and methods such as “ROR, PRR, BCPNN, and MGPS” were adopted for ADE signals detection. The criteria for determining valid ADE signals were set as follows: number of ADE reports ≥ 3, lower limit of 95% CI for ROR > 1, PRR ≥ 2, *χ*^2^ ≥ 4, IC_025_ > 0, EBGM_05_ > 2, and a > 0. When conducting ADE signal detection at the PT level based on the FAERS database, the same methods of “ROR, PRR, BCPNN, and MGPS” were still used, and the criteria for determining valid signals remained consistent. In addition, the “ggplot2” software package (v 3.5.1) (19) was used to visually present the results at both the SOC level and the PT level.

### 2.5 Analysis of gender, age, and time-to-onset related to MTX-induced ADEs in PL treatment using FAERS data

To characterize sex-related differences in the reporting patterns of MTX-associated ADEs in PL, data were extracted from ADE reports related to MTX for PL treatment in the FAERS database spanning from the first quarter of 2018 to the first quarter of 2025. A stratified analysis by gender was conducted, where data for male and female patients were extracted separately, and the ROR was recalculated. An ROR with a lower bound of the 95% CI greater than 1 indicated that ADE reports for the relevant drug were more frequent, suggesting a potential safety signal. The “forestplot” package (v 1.1.1) (20) was used to generate a forest plot of PTs with an ROR lower bound of 95% CI greater than 1.

To visually demonstrate the safety differences in MTX-related ADEs, four algorithms—ROR, PRR, BCPNN, and MGPS—were employed for ADE signal detection in the signal detection process for ADE reports related to MTX for PL treatment in the FAERS database from the first quarter of 2018 to the first quarter of 2025. The criteria for determining valid signals were as follows: the number of ADE reports ≥ 3, ROR lower bound of 95% CI > 1, PRR ≥ 2, *χ*^2^ ≥ 4, IC_025_ > 0, EBGM_05_ > 2, and a > 0. Only PTs that met the criteria of all four algorithms were identified as valid ADE signals, and visualization was performed using the “ggplot2” package (v 3.5.1).

To compare the distribution characteristics of ADEs across different age groups, the pediatric population was divided into five age groups: infants (<1 year), toddlers (1–3 years), preschoolers (4–6 years), school-age children (7–10 years), and adolescents (11–17 years). Data on ADE reports related to MTX for PL treatment were extracted for each age group separately from the FAERS database from the first quarter of 2018 to the first quarter of 2025, and the top 20 PTs by the number of reports were selected for each group. Bubble plots were generated using the “ggplot2” package (v 3.5.1) for visualization. Additionally, an intersection analysis was conducted on the valid ADE signals across the five age groups to identify common PTs present in all age groups, further characterizing age-stratified reporting patterns of MTX-associated ADEs.

To further explore the time-to-onset of ADEs related to MTX for PL treatment, for the time-to-onset analysis, statistical analysis was performed based on the time-to-onset (TTO) of ADEs recorded in the FAERS database from the first quarter of 2018 to the first quarter of 2025.For the TTO analysis, the time to adverse event was calculated based on the available treatment start date and adverse event occurrence date. After excluding reports with missing or unreliable onset dates (e.g., missing START_DT or EVENT_DT, or implausible TTO values), a total of 944 reports were included in the TTO analysis, accounting for 32.5% of the overall 2,907 reports. Reports lacking necessary date information or for which the TTO could not be reliably calculated were excluded from the TTO analysis. The TTO was ultimately categorized into the following intervals for analysis: 0–30, 31–60, 61–90, 91–120, 121–150, 151–180, 181–360, and >360 days. This described the temporal distribution of reported events and informed potential pharmacovigilance windows. To further explore the time-to-onset characteristics of MTX-related ADEs in PL, we additionally performed survival analysis using the R package “survival” (v 3.7.0) (21) and plotted TTO cumulative curves with “survminer” (v 0.4.9) (22). The “fitdistrplus” package (v 1.1.11) (23)was used to fit a Weibull distribution and estimate the Weibull shape parameter (WSP, *β*) with its 95% CI. Classification was defined as: early failure (*β* < 1% and 95% CI < 1, decreasing hazard over time), random failure (*β* = 1% and 95% CI = 1, constant hazard), and wear-out failure (*β* > 1% and 95% CI > 1, increasing hazard). Leukemia subtype, risk-stratification group, treatment phase, MTX dose or intensity, cumulative exposure, supportive care, and concomitant medications were not included as adjustment variables because these data were unavailable or incompletely reported in FAERS.

### 2.6 Statistical analysis

The association between MTX and PL in PL treatment was analyzed using the ROR, PRR, BCPNN, and MGPS algorithms. In this study, R packages (v 4.3.1) were used for statistical analysis and data processing.

## 3 Results

### 3.1 The basic characteristics of MTX treatment for PL

Based on the statistical results of 2,907 ADE reports related to MTX for PL treatment extracted from the FAERS database from the first quarter of 2018 to the first quarter of 2025, the following findings were revealed: In terms of gender, male patients accounted for the highest proportion of reports (58.3%), while female patients accounted for 38.3%. Regarding body weight, reports involving patients with a body weight of 10–20 kg accounted for the highest proportion (13.24%), and those involving patients with a body weight of 21–40 kg accounted for 12.11%. In terms of age, adolescents aged 11–17 years accounted for the highest proportion of reports (37.8%), and the proportions of reports for children aged 4–6 years and 7–10 years were the same (both 21.7%). For outcomes, ADEs resulting in hospitalization (HO) accounted for the highest proportion (41.9%), and those posing a life-threatening risk (LT) accounted for 14.9%. In terms of reporter type, healthcare professionals (HP) and physicians (MD) stood out with proportions of 39.3% and 36.5% respectively. Regarding the reporting countries, the reports covered 57 countries in total, among which the US had the highest proportion (20.9%), followed by Canada (CA) at 17.7%, France (FR) at 15.0%, China(CN) at 9.5%,Italy (IT) at 7.1%. These descriptive findings indicate that reports involving males and adolescents aged 11–17 years were more frequent in the analyzed dataset; they do not demonstrate higher incidence or clinical risk because FAERS does not provide exposure denominators. Meanwhile, experience from European and American countries in reporting could be referenced to further improve the ADE monitoring and reporting system (Table 2).

**Table 2:** The characteristics of ADE reports pertaining to MTX treatment for PL.

Characteristics	(*N*,%)
Total number of reports	(*N* = 2,907)
Sex	
Female	1,114 (38.3%)
Male	1,695 (58.3%)
missing	98 (3.4%)
Weight	
＜10 kg	68 (2.3%)
10～20 kg	385 (13.2%)
21～40 kg	352 (12.1%)
41～60 kg	213 (7.3%)
＞60 kg	96 (3.3%)
missing	1,793 (61.7%)
Age (years)	
<1	66 (2.3%)
1–3	482 (16.6%)
4–6	630 (21.7%)
7–10	630 (21.7%)
11–17	1,099 (37.8%)
^a^Occupation Code	
Consumer (CN)	160 (5.5%)
Healthcare professional (HP)	1,141 (39.3%)
Lawyer (LW)	2 (0.1%)
Physician (MD)	1,062 (36.5%)
Other health-professional (OT)	317 (10.9%)
Pharmacist (PH)	211 (7.3%)
Missing	14 (0.5%)
Outcome Code	
Death (DE)	202 (6.9%)
Disability (DS)	26 (0.9%)
Hospitalized (HO)	1,218 (41.9%)
Life-Threatening (LT)	432 (14.9%)
Other Serious (OT)	1,023 (35.2%)
Missing	6 (0.2%)
Reporter Country	
United States (US)	608 (20.9%)
Canada (CA)	515 (17.7%)
France (FR)	436 (15.0%)
China (CN)	275 (9.5%)
Italy (IT)	207 (7.1%)
Spain (ES)	130 (4.5%)
Japan (JP)	90 (3.1%)
Germany (DE)	76 (2.6%)
Argentina (AR)	34 (1.2%)
United Kingdom (GB)	34 (1.2%)

a Occupation code refers to the occupation of the adverse-event reporter submitting the FAERS report, not the occupation of the patient.

### 3.2 Analysis of MTX-associated ADE signals in PL (SOC & PT levels)

When conducting a SOC-level analysis of ADE signals associated with MTX in the treatment of PL, a total of 26 SOC levels were found to have MTX-related ADE signals for PL treatment. Among these, 7 were valid signals that met the criteria, specifically as follows: valid ADE signals meeting the criteria were present in the categories of hepatobiliary disorders, gastrointestinal disorders, nervous system disorders, psychiatric disorders, skin and subcutaneous tissue disorders, renal and urinary disorders, and eye disorders. Among these categories, the nervous system disorders category had the highest number of ADE reports, reaching 1,691 cases, with a ROR (95% CI) of 2.87 (2.69–3.05), a PRR of 2.53, a *χ*^2^ value of 1,126.79, and an IC_025_ of 0.92. These valid signals that met the criteria indicated that during the clinical use of MTX for PL treatment, close clinical monitoring is required for potential adverse events in children related to the hepatobiliary system, gastrointestinal tract, nervous system, psychiatric status, skin and subcutaneous tissues, renal and urinary systems, and eyes. In particular, attention should be paid to the occurrence of ADEs related to nervous system disorders, so as to provide a targeted monitoring direction for ensuring the medication safety of children (Figure 1).

**Figure 1:**
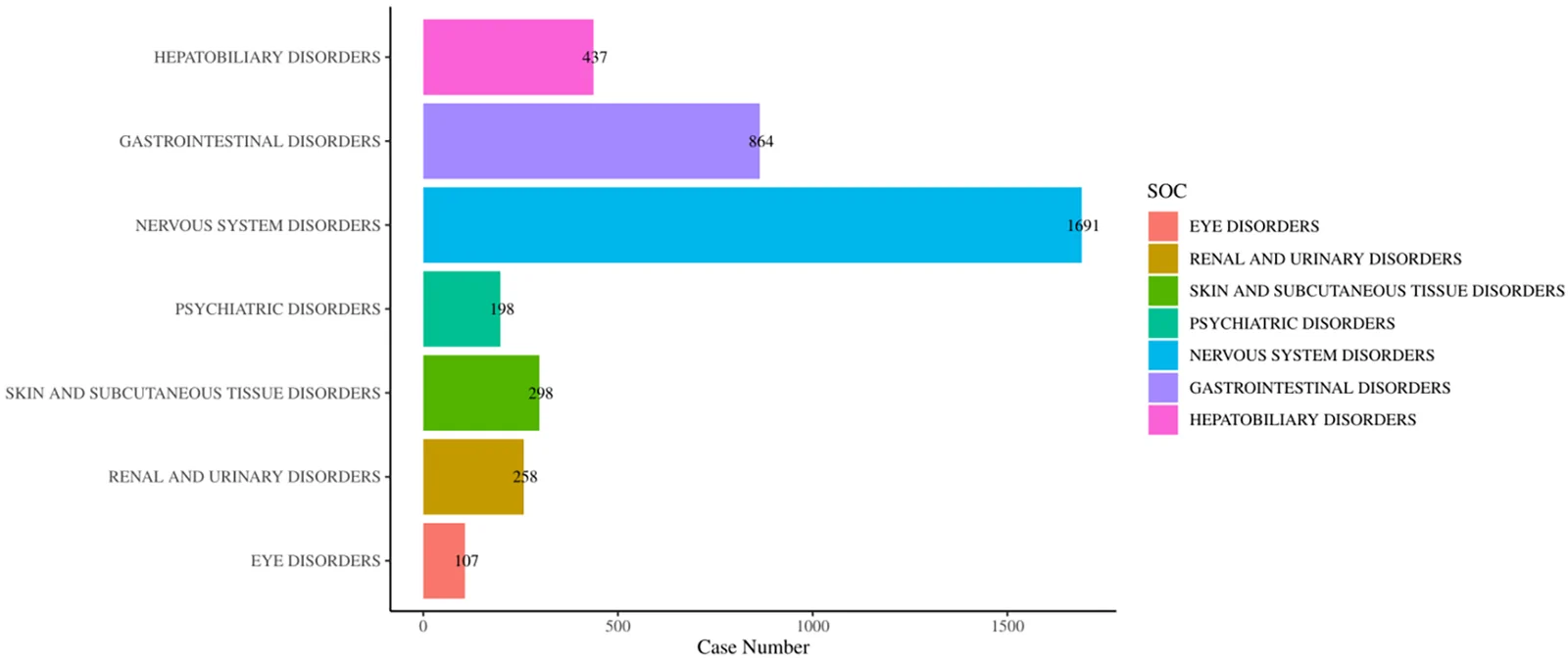
ADE reports across 7 valid SOC categories associated with MTX use in PL.

In the analysis of MTX for PL, a total of 1,280 ADE signals at the PT level were involved. After evaluation, 274 valid signals were identified, and these valid signals corresponded to 15 SOCs. Among them, febrile neutropenia had the highest number of reports, reaching 446 cases, with a ROR (95% CI) of 1.44 (1.29–1.6), a *χ*^2^ value of 44.27, and an IC_025_ of 0.25; followed by neurotoxicity, with 238 reports; and mucosal inflammation came next, with 170 reports. These three were the most frequently reported PTs in the analyzed MTX-associated reports (Figure 2). These reporting patterns suggest that the three PTs warrant attention in pharmacovigilance (Supplementary Table S2).

**Figure 2:**
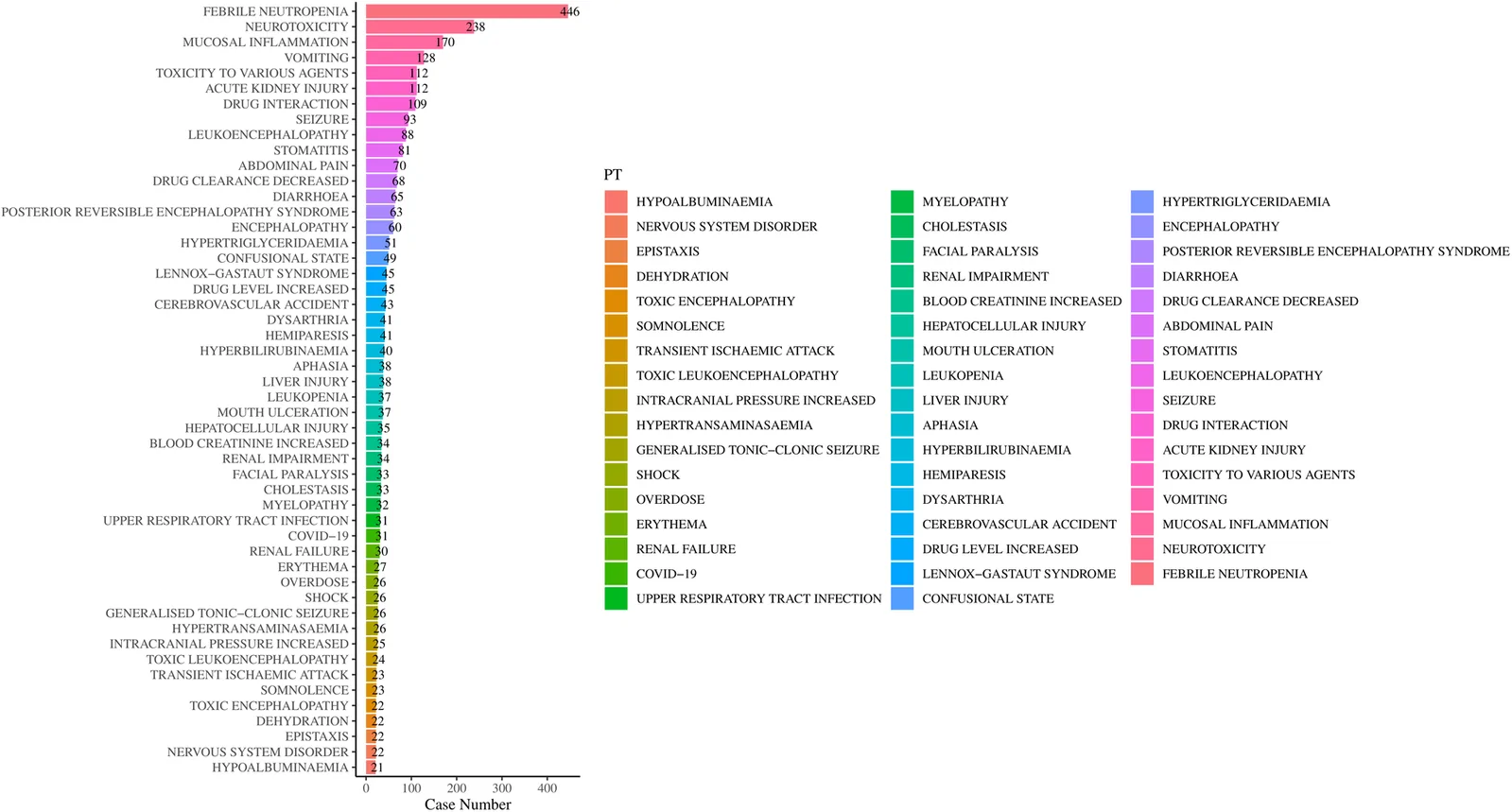
ADE reports at the PT level associated with MTX use in PL.

### 3.3 Results of gender subgroup analysis of ADEs related to MTX in the treatment of PL

In the gender subgroup analysis of ADEs related to MTX in the treatment of PL, a total of 149 gender-related ADE signals were detected, among which 12 PTs showed potential safety signals in different gender subgroups. Specifically, there were 40 reports of confusional state in males and 9 in females, with a ROR (95% CI) of 2.79 (1.35–5.76); there were 20 reports of dehydration in males and 1 in females, with a ROR (95% CI) of 12.55 (1.68–93.56); and there were 20 reports of epistaxis in males and 2 in females, with a ROR (95% CI) of 6.27 (1.47–26.86). These results indicate greater reporting frequency and/or disproportionality for these PTs among male reports, more attention could be paid to the occurrence of confusional state, dehydration, and epistaxis in male children based on these reporting signals to achieve more targeted pharmacovigilance monitoring. These sex-related differences describe reporting disproportionality within the FAERS database only. Because FAERS lacks sex-specific exposure denominators, detailed treatment information, and complete clinical context, these findings cannot be interpreted as higher incidence, greater clinical risk, or increased biological susceptibility in males. They should be regarded as hypothesis-generating pharmacovigilance signals requiring independent validation in prospective, exposure-adjusted studies. (Figure 3; Supplementary Table 3).

**Figure 3:**
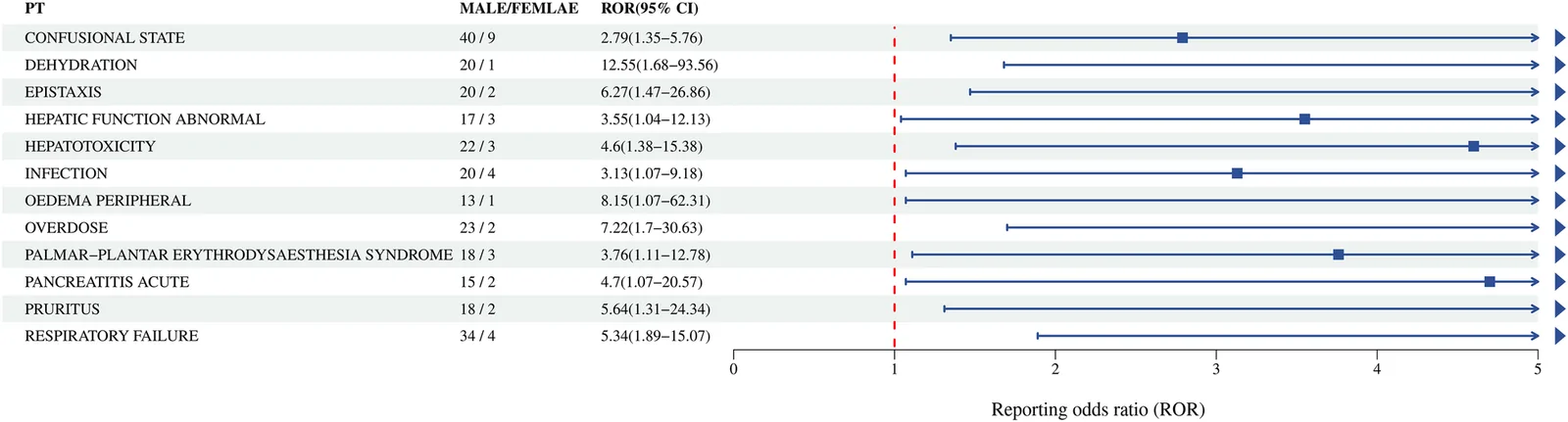
Forest plot of ROR for ADEs at the PT level, stratified by gender in PL treated with MTX.

### 3.4 Results of ADE signal detection and age-stratified analysis of MTX in the treatment of PL

In the ADE signal detection of MTX for PL treatment, among 1280 PTs, 16 PTs simultaneously met the valid signal criteria of four algorithms: ROR, PRR, BCPNN, and MGPS. The results of the bubble chart showed that LENNOX-GASTAUT syndrome exhibited positive reporting disproportionality [log(ROR) > 0] and a high reporting proportion, suggesting that the association between this nervous system-related adverse reaction and MTX required focused attention; while neurotoxicity had the largest number of ADE reports, reflecting its prominence among FAERS reports rather than its clinical prevalence (Figure 4A).

**Figure 4:**
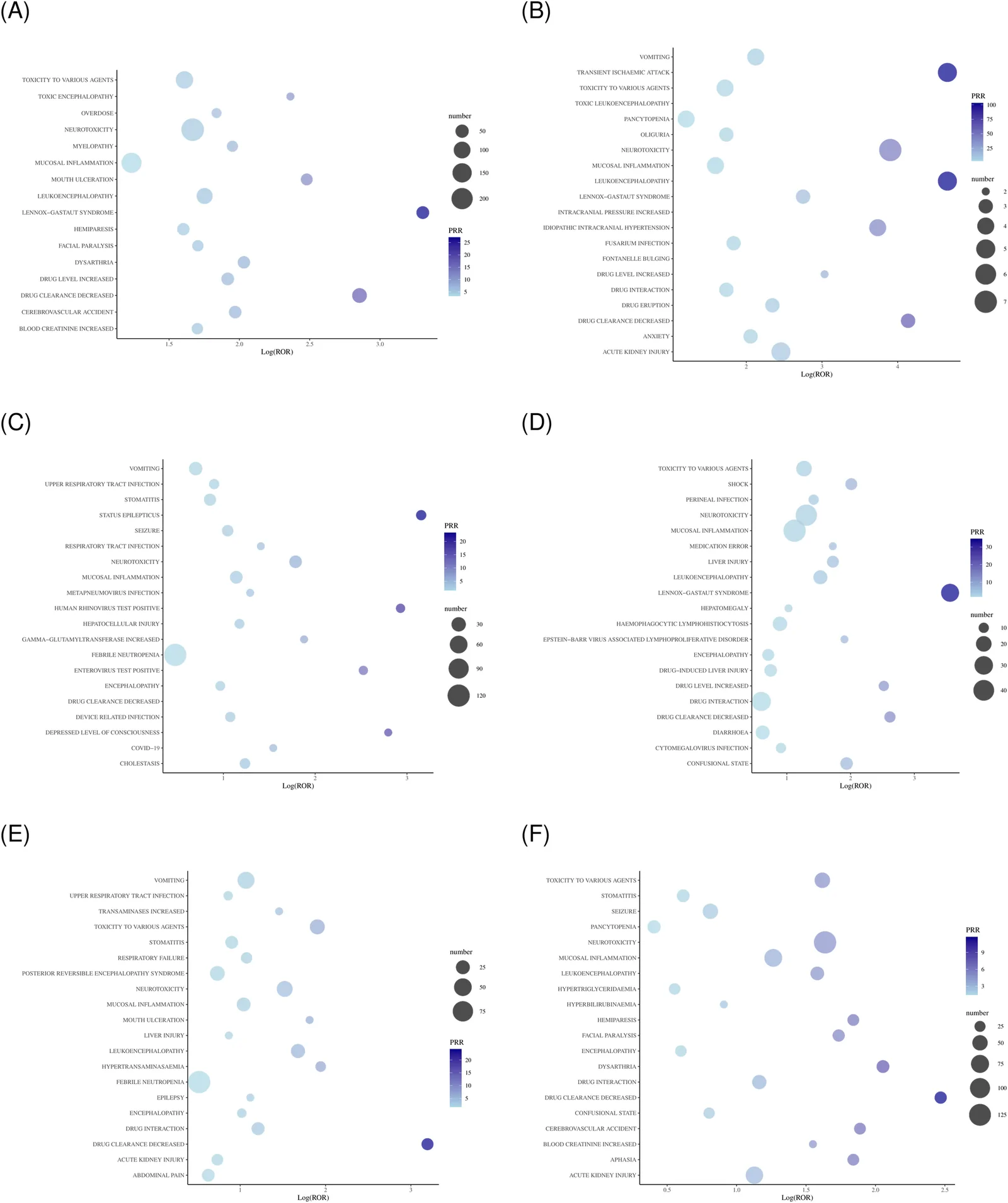
Bubble chart of ADE signal detection and age-stratified analysis for MTX in the treatment of PL. Bubble chart of ADE signal detection and age-stratified analysis for MTX in the treatment of PL. **(A)** ADE signals for MTX in the overall PL patient cohort. **(B)** The top 20 ADE PTs in infants (<1 year old) with PL treated with MTX. **(C)** The top 20 ADE PTs in toddlers (1−3 years old) with PL treated with MTX. **(D)** The top 20 ADE PTs in Preschoolers (4−6 years old) with PL treated with MTX. **(E)** The top 20 ADE PTs in School-age children (7−10 years old) with PL treated with MTX. **(F)** The top 20 ADE PTs in Adolescents (11−17 years old) with PL treated with MTX.

In the age-stratified analysis, infants (<1 year old), toddlers (1–3 years old), preschoolers (4–6 years old), school-age children (7–10 years old), and adolescents (11–17 years old) corresponded to 21, 77, 94, 101, and 139 PTs, respectively. The leading PT by report count differed across age groups: neurotoxicity was the leading PT in infants and adolescents, febrile neutropenia in toddlers and school-age children, and mucosal inflammation in preschoolers. Six PTs—neurotoxicity, leukoencephalopathy, mucosal inflammation, toxicity to various agents, drug clearance decreased, and drug level increased—were detected in all five age strata. These findings describe age-specific reporting patterns and cross-age signals only. Because FAERS lacks exposure denominators and detailed treatment information, the observed differences may reflect unequal MTX exposure, leukemia subtype, risk stratification, treatment phase or intensity, supportive care, concomitant therapy, or reporting behavior rather than true age-related differences in incidence or susceptibility (Figures 4B–F; Supplementary Table S4). The age-stratified differences should therefore be regarded as hypothesis-generating signals that require prospective, controlled evaluation.

### 3.5 Analysis of onset time

Based on the FAERS data from the first quarter of 2018 to the first quarter of 2025, the onset time of ADE of MTX in the treatment of PL was investigated. Among the 944 reports with evaluable onset data, 681 cases (72.1%) occurred within 0–30 days after medication, representing the largest proportion; 89 cases (9.4%) occurred within 31–60 days; 30 cases (3.2%) within 61–90 days; 20 cases (2.1%) within 91–120 days; 6 cases (0.6%) within 121–150 days; 15 cases (1.6%) within 151–180 days; 59 cases (6.2%) within 181–360 days; and 44 cases (4.7%) beyond 360 days. Overall, the reported onset-time distribution for MTX was concentrated in the first 30 days, representing a temporal reporting signal for further investigation rather than a causal risk window (Figure 5A). Survival analysis further revealed that the median time to onset of MTX-related ADEs was 11 days. This temporal reporting pattern reflects the distribution of submitted reports rather than a time-specific incidence peak or causal risk window. The observed decline in reporting frequency after 30 days may be influenced by treatment phase transitions, follow-up duration, reporting behavior, and concomitant therapy, and should not be interpreted as diminishing hazard over time. Weibull distribution fitting showed that the WSP estimate *β* was less than 1 with the entire 95% CI below 1, indicating an early reporting pattern (i.e., decreasing reporting frequency over time in the observed dataset). (Figure 5B; Table 3). These analyses describe the timing of submitted reports and do not estimate a time-specific incidence or hazard of events causally attributable to MTX.

**Figure 5:**
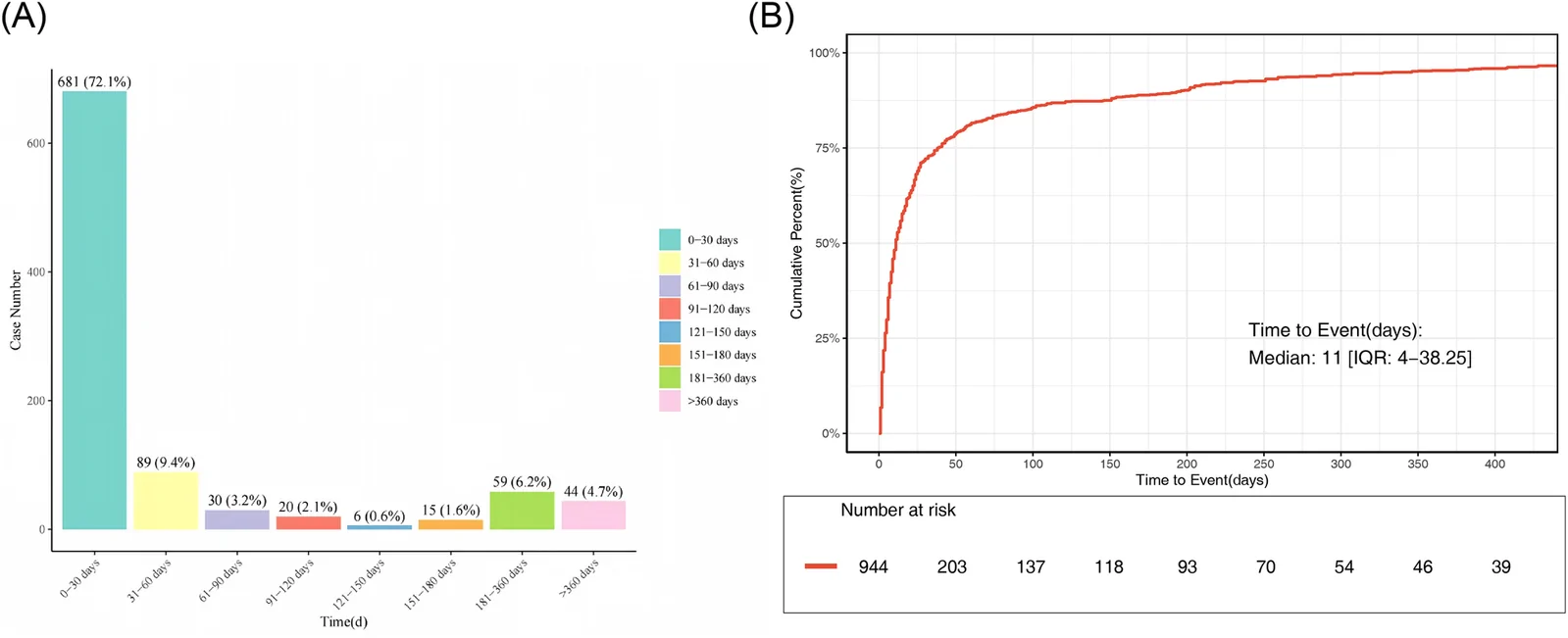
Onset time analysis of MTX-associated ADEs in PL treatment. **(A)** Onset time distribution of MTX-associated ADE reports in PL treatment. **(B)** Time-accumulation curve of MTX-associated ADE reporting.

**Table 3:** Weibull shape parameter test.

Drug	Median (days)	Scale parameter, alpha	Shape parameter, beta	Reporting Pattern
Methotrexate	11 (4–50)	37.9 (35.15–40.65)	0.55 (0.53–0.56)	Early reporting pattern (decreasing hazard over time)

## 4 Discussion

PL is a malignant clonal disorder of bone marrow hematopoietic stem cells (24). MTX is a key chemotherapeutic drug that inhibits the folate metabolic pathway and targets rapidly proliferating leukemia cells (25). However, it lacks cell specificity and can damage normal proliferating tissues, resulting in a variety of adverse reactions (15, 26). Based on the FAERS database, this study systematically identified MTX-related ADE signals and further analyzed sex-, age-, and time-stratified reporting patterns, providing hypothesis-generating pharmacovigilance evidence on MTX-associated ADE reports in PL.

This study found that ADE reports were most frequent among males and adolescents aged 11–17. Because FAERS lacks sex- and age-specific exposure denominators, these proportions cannot be interpreted as higher incidence or clinical risk and may be influenced by leukemia subtype, treatment phase or intensity, concomitant therapy, supportive care, and reporting behavior. This distribution may be related to factors such as drug metabolism and disease biology. Previous studies have suggested that gender differences in metabolic enzymes and transporters may influence MTX detoxification and accumulation (27, 28). Additionally, active bone marrow hematopoiesis, intensive treatment regimens, and higher MTX dosages in some adolescents may provide possible explanations for the observed neurotoxicity and myelosuppression reporting patterns (29, 30). Furthermore, high-dose MTX can penetrate the relatively mature blood-brain barrier (BBB), inducing central nervous system (CNS) toxicity (31), which may provide biological plausibility for the reporting pattern but does not establish increased incidence in this subgroup.

At the SOC level, the ADE reports for neurological diseases were the most numerous, with an ROR (95% CI) of 2.87 (2.69–3.05). This reporting signal cannot establish that MTX caused the reported neurological events; the mechanisms discussed below provide biological plausibility only. MTX can enter the CNS via two pathways: passive diffusion across the BBB as plasma drug concentrations rise (particularly at high doses), and active transport through the choroid plexus (13). Once in the CNS, MTX inhibits dihydrofolate reductase (DHFR) in neurons, leading to folate deficiency that impairs synthesis of neurotransmitters such as dopamine and serotonin, potentially causing cognitive impairment and confusion (31, 32). Concurrently, insufficient tetrahydrofolate inhibits myelin formation, causing myelin damage and white matter lesions (28). Additionally, MTX activates microglia in the CNS, promoting the release of inflammatory factors such as TNF-α and IL-6, thereby triggering neuroinflammation (32, 33). These inflammatory factors can directly damage nerve cells. They also destroy the integrity of the BBB, thereby increasing the amount of MTX that enters the CNS, forming a “toxic-inflammatory” vicious cycle that further exacerbates neurological damage.

At the PT level, febrile neutropenia had the highest report count, followed by neurotoxicity and mucositis. This reporting prominence cannot be attributed to MTX alone because pediatric leukemia protocols commonly use multi-agent chemotherapy; MTX-related bone marrow suppression is one plausible contributor. MTX inhibits bone marrow hematopoiesis. Hematopoietic stem cells(HSCs) are the source of neutrophils, and their proliferation and differentiation depend on continuous DNA synthesis. MTX inhibits DHFR in HSCs, resulting in a lack of tetrahydrofolate and causing HSCs to become trapped in the S phase, preventing them from differentiating into directed hematopoietic progenitor cells (34, 35). MTX can also induce HSC apoptosis by activating the p53 pathway and increasing the expression of pro-apoptotic proteins,which promotes HSC death (36, 37). The combined effects of suppressed proliferation and increased apoptosis in HSCs lead to reduced neutrophil production. In addition to decreased numbers, MTX also impairs neutrophil function. Neutrophils' phagocytic ability and respiratory burst require ATP. However, folate deficiency inhibits glycolysis and oxidative phosphorylation pathways, resulting in decreased ATP production and impaired neutrophil function (38, 39). Dysfunctional neutrophils cannot effectively eliminate pathogens (40, 41). Additionally, the reporting prominence of these ADEs may also reflect the PL treatment regimen-the induction phase often involves the use of MTX and other chemotherapeutic agents,which may produce overlapping toxicities and preclude attribution of these events to MTX alone (42, 43).

Confusional state, dehydration, and epistaxis showed stronger reporting among male reports; FAERS cannot determine whether these differences reflect biology, exposure, treatment, or reporting, and the following mechanistic explanations remain hypotheses. The occurrence of impaired consciousness is associated not only with lower GST*μ*1 expression and MDR1 activity in males leading to central accumulation of MTX, but also involves neuropharmacological mechanisms: MTX inhibits gamma-aminobutyric acid (GABA) transaminase, reducing GABA degradation and thereby increasing GABA concentration. Males exhibit higher expression of the GABA receptor *α*2 subtype, making them more sensitive to elevated GABA levels and thus more prone to developing confusion (44–46). The reported dehydration signal may have several possible physiological explanations. MTX increases urine output by inhibiting water reabsorption in renal tubules through its effect on aquaporin 2 (AQP2) expression in tubular epithelial cells (47). Concurrently, males' higher basal metabolic rates (BMR), greater physical activity, increased fluid requirements and losses, and testosterone's mild suppression of antidiuretic hormone (ADH), which promotes water retention, may contribute to the reported dehydration signal (48). The epistaxis reporting difference may have possible links to coagulation and vascular effects: MTX inhibits the proliferation and differentiation of bone marrow megakaryocytes, leading to thrombocytopenia (17), while also suppressing the synthesis of the protective vasodilator prostaglandin I2 (PGI2) in vascular endothelium. Male endothelial cells exhibit greater sensitivity to MTX inhibition, resulting in more pronounced reductions in PGI2 synthesis and increased vascular fragility (49, 50). This synergizes with thrombocytopenia to elevate bleeding risk. These proposed sex-specific mechanisms cannot be established from FAERS and require independent validation.

Differences in the primary ADE reporting patterns across pediatric age groups may reflect developmental factors but may also arise from differences in exposure and treatment; the mechanisms discussed below are therefore hypothetical. Infants (<1 year) exhibit an underdeveloped BBB and lower expression levels of tight junction proteins, resulting in high BBB permeability (29). Consequently, MTX readily crosses the immature BBB into the central nervous system. Additionally, infants possess underdeveloped hepatic and renal function with limited clearance capacity, facilitating MTX entry and accumulation in the central nervous system. Combined with their active neural cell proliferation and extreme sensitivity to folate inhibition, these features may contribute to the observed neurotoxicity reporting pattern, although incidence cannot be inferred from FAERS (51–54). In toddlers (1–3 years) and school-aged children (7–10 years), bone marrow hematopoietic function is relatively more active, which may contribute to the reported prominence of neutropenia. During these two age groups, hematopoietic cell proliferation is rapid, making them more sensitive to MTX's cytotoxic effects (16). Consequently, bone marrow suppression and associated febrile neutropenia may be prominent in reports, although multi-agent treatment may also contribute. In preschool children (4–6 years), mucosal tissues remain immature with limited epithelial repair capacity. Following mucosal injury, they are more susceptible to bacterial infections, which exacerbate inflammatory responses and lead to mucosal inflammation (55, 56). Despite these age-specific differences, six reporting signals, including neurotoxicity and leukoencephalopathy,were detected across age strata. These cross-age signals may be biologically compatible with MTX-related folate inhibition, a fundamental cellular process across all age groups that relies on folate for nucleic acid and neurotransmitter synthesis (57, 58). Furthermore, events like leukoencephalopathy are also associated with drug-induced vascular endothelial injury and subsequent ischemia-hypoxia (59, 60); however, this mechanism cannot be inferred from the FAERS analysis.

Among reports with available onset data, the largest proportion described events within 0–30 days (72.1%); this is a temporal reporting pattern rather than an incidence peak and may also reflect treatment phase, concomitant therapy, follow-up, or reporting behavior. During the induction phase of PL treatment, which typically occurs in the first 1–2 months, high-dose exposure is often used to rapidly reduce the burden of leukemia cells. High-dose MTX may increase toxicity to normal cells and extend the drug's residence time in the body (61, 62), providing biological plausibility for early reports but not establishing a causal time-specific risk. The early reporting cluster may be influenced by treatment phase, dosing schedule, or repeated exposure; however, FAERS did not provide sufficiently complete cycle-level dosing information to determine whether events followed biweekly, monthly, or other methotrexate schedules.

Regarding specific adverse events, mucosal inflammation typically occurs within one week due to the short epithelial cell renewal cycle. Febrile neutropenia commonly manifests 2–3 weeks post-treatment as neutrophil counts reach their lowest point (63, 64). Neurotoxicity encompasses both acute reactions occurring 24–48 h after administration and subacute reactions emerging 1–2 weeks later, both falling within the 30-day window. Fewer reports described onset after 30 days; this should not be interpreted as declining incidence. The pattern may reflect treatment phase, concomitant therapy, follow-up, or reporting behavior, while cumulative or delayed toxicities may remain relevant (65).

Based on these findings, future research could evaluate whether these reporting signals can inform monitoring strategies for MTX-associated ADEs in PL. Sex-, age-, and time-specific surveillance should follow established clinical protocols and be assessed prospectively rather than inferred directly from FAERS subgroup proportions. These hypothesis-generating signals warrant validation in prospective, controlled studies with exposure denominators and detailed regimen data.

This study has several limitations. First, FAERS is a spontaneous reporting system subject to under-reporting, stimulated reporting, duplicate or incomplete information, and other reporting biases. It does not provide the number of patients exposed to MTX, person-time, treatment duration, or a representative sampling frame; therefore, reporting proportions and disproportionality metrics cannot be used to estimate incidence, frequency, absolute risk, or comparative clinical risk (66). Second, pediatric leukemia was analyzed as a single cohort even though MTX dose, route, schedule, cumulative exposure, treatment intensity, supportive care, and co-administered agents vary by leukemia subtype, risk-stratification group, and induction, consolidation, or maintenance phase. These variables were unavailable or insufficiently complete in FAERS and could not be controlled, creating substantial treatment heterogeneity and confounding. Third, concomitant medications were not systematically evaluated because drug records and clinical context were incomplete. Many events, particularly febrile neutropenia, mucositis, neurotoxicity, and hepatotoxicity, may reflect leukemia itself, multi-agent chemotherapy, supportive medications, infections, or interactions rather than MTX alone. Consequently, FAERS cannot distinguish the contribution of MTX from that of combination therapy or establish causality. Fourth, residual confounding from demographic, clinical, genetic, and reporting factors remains. Next, limited follow-up restricts assessment of delayed or chronic toxicities, and the reporting population may not represent all treatment settings. Then, because quantitative methotrexate concentrations and sampling times were unavailable or incompletely reported, relationships between serum methotrexate levels and specific ADEs could not be evaluated. Finally, dosing frequency, number of administrations, cycle number, and cumulative exposure could not be reliably evaluated; therefore, the 0–30-day reporting pattern cannot be attributed to a specific dosing schedule or repeated exposure. The mechanistic explanations and subgroup findings should therefore be regarded as hypothesis-generating and validated in prospective, exposure-adjusted studies.

## 5 Conclusion

In conclusion, this FAERS analysis identified MTX-associated reporting patterns and disproportionality signals in pediatric leukemia. Nervous system disorders generated prominent SOC-level signals, while febrile neutropenia, neurotoxicity, and mucositis were among the most frequently reported PTs. Reports involving males and adolescents constituted larger proportions, and reports with available onset data were concentrated within 30 days. The observed sex-, age-, and time-related differences should be interpreted as potential pharmacovigilance signals rather than confirmed clinical associations. They do not demonstrate higher incidence in any subgroup or establish that MTX caused the reported events, particularly given treatment heterogeneity and concomitant multi-agent therapy. These findings are hypothesis-generating and require evaluation in prospective, controlled studies with exposure denominators, detailed leukemia subtype and treatment-phase data, cumulative dosing, concomitant medications, and external validation.

## Data Availability

Publicly available datasets were analyzed in this study. These data can be found in the US Food and Drug Administration Adverse Event Reporting System (FAERS) Quarterly Data Extract Files at https://fis.fda.gov/extensions/FPD-QDE-FAERS/FPD-QDE-FAERS.html.
