# Supplementary material for: Sex- and age-stratified pharmacovigilance of methotrexate-associated adverse events in pediatric leukemia: a FAERS-based study
Source: Front Med (Lausanne). 2026 Sep 28;13:1948388. doi: 10.3389/fmed.2026.1948388 (PMC13654699; doi:10.3389/fmed.2026.1948388)
Supplement: Supplementary file 1 [file Table1.docx]

**Supplementary Table 1** Four formulas for imbalance methods

| Method | Formula | Threshold |
| --- | --- | --- |
| ROR | $ROR = \frac{a/c}{b/d}$ | $a\geq\left( lower limit \right)>1$ |
|  | $\mathrm{SE}\left( \mathrm{lnROR} \right)=\sqrt{\frac{1}{a}+\frac{1}{b}+\frac{1}{c}+\frac{1}{d}}$ |  |
|  | $95\%CI = e^{\ln\left( \mathrm{ROR} \right)\pm1.96SE\left( \mathrm{lnROR} \right)}$ |  |
| PRR | $PRR=\frac{a/\left( a+b \right)}{c/{（c+d）}}$ | $a\geq3 PRR \geq2$ and $\chi^{2}>4$ |
|  | $\chi^{2}=\frac{{(ad - bc)}^{2}(a + b + c + d)}{(a + b)(c + d)(a + c)(b + d)}$ |  |
| BCPNN | $IC = \log_{2}\frac{a\left( a+b+c+d \right)}{\left( a+b \right)\left( a+c \right)}$ | IC_025_>0 |
|  | $E\left( \mathrm{IC} \right) = \log_{2}\frac{\left( a+1 \right)\left( a+b+c+d+\alpha\right)\left( a+b+c+d+\beta\right)}{\left( a+b+c+d+\gamma\right)\left( a+b+\alpha\right)\left( a+c+\beta\right)}$ |  |
|  | $V\left( \mathrm{IC} \right)=\frac{1}{\left( ln 2 \right)^{2}}\left[ \frac{\left( a+b+c+d \right)-a+\gamma-11}{\left( a+\gamma11 \right)\left( 1+a+b+c+d+\gamma\right)}+\frac{\left( a+b+c+d-\left( a+b \right)+a-\alpha1 \right)}{\left( a+b+\alpha1 \right)\left( 1+a+b+c+d+\alpha\right)}+\frac{\left( a+b+c+d+\alpha\right)-\left( a+c \right)+\beta-\beta1}{\left( a+b+\beta1 \right)\left( 1+a+b+c+d+\beta\right)} \right]$ |  |
|  | $\gamma= \gamma11\frac{\left( a+b+c+d+\alpha\right)\left( a+b+c+d+\beta\right)}{\left( a+b+\alpha1 \right)\left( a+c+\beta\right)}$ |  |
|  | $IC-2SD = E\left( \mathrm{IC} \right)-2\sqrt{V\left( \mathrm{IC} \right)}$ |  |
| MGPS | $\mathrm{EBGM} = \frac{a\left( a+b+c+d \right)}{\left( a+c \right)\left( a+b \right)}$ | EBGM_05_ > 2 and a > 0 |
|  | EBGM_05 =_ $e^{\ln\left( \mathrm{EBGM} \right)-2\sqrt{1.64(\frac{1}{a}+\frac{1}{b}+\frac{1}{c}+\frac{1}{d})}}$ |  |
